# Distinct Functions for Different *scl* Isoforms in Zebrafish Primitive and Definitive Hematopoiesis

**DOI:** 10.1371/journal.pbio.0050132

**Published:** 2007-05-01

**Authors:** Feng Qian, Fenghua Zhen, Jin Xu, Mei Huang, Wanyu Li, Zilong Wen

**Affiliations:** 1 Laboratory of Molecular and Developmental Immunology, Institute of Molecular and Cell Biology, Singapore, Singapore; 2 Department of Biological Sciences National University of Singapore, Singapore, Singapore; Baylor College of Medicine, United States of America

## Abstract

The stem-cell leukemia (SCL, also known as TAL1) gene encodes a basic helix-loop-helix transcription factor that is essential for the initiation of primitive and definitive hematopoiesis, erythrocyte and megakarocyte differentiation, angiogenesis, and astrocyte development. Here we report that the zebrafish produces, through an alternative promoter site, a novel truncated *scl (tal1)* isoform, *scl-*β, which manifests a temporal and spatial expression distinct from the previously described full-length *scl-*α. Functional analysis reveals that while *scl-*α and -β are redundant for the initiation of primitive hematopoiesis, these two isoforms exert distinct functions in the regulation of primitive erythroid differentiation and definitive hematopoietic stem cell specification. We further demonstrate that differences in the protein expression levels of *scl-*α and -β, by regulating their protein stability, are likely to give rise to their distinct functions. Our findings suggest that hematopoietic cells at different levels of hierarchy are likely governed by a gradient of the Scl protein established through temporal and spatial patterns of expression of the different isoforms.

## Author Summary

Hematopoiesis is the process that generates all the body's blood cells. In vertebrates, hematopoietic development occurs in two phases: a transitory embryonic, or primitive, wave produces only erythrocytes (red blood cells) and myeloid cells; an adult, or definitive, wave gives rise to at least three blood cell lineages, including erythrocytes and two types of immune cells—myeloid cells and lymphocytes. Previous studies have shown that the stem-cell leukemia (SCL) gene is essential for hematopoietic stem cell specification and erythrocyte maturation**.** Yet how SCL regulates these distinct processes is not fully understood. This study demonstrates that zebrafish produce a smaller isoform of *scl, scl-*β, that plays an overlapping role with the full-length *scl-*α in the initiation of primitive hematopoiesis, but possesses distinct functions in regulating primitive erythrocyte maturation and definitive hematopoietic stem cell development. We further show that the distinct functions of *scl-*α and -β are likely due to differences in their protein expression level through the regulation of protein stability. We postulate that hematopoietic cell development at different levels of hierarchy is governed by a gradient of the Scl protein created by temporal and spatial expression of different *scl* isoforms.

## Abstract 3

The authors show that the single vertebrate SCL gene produces two separately controlled transcripts with differing effects on zebrafish hematopoiesis.

## Introduction

On the basis of anatomic locations of development, time of initiation, and cell type produced, vertebrate hematopoiesis can be divided into primitive and definitive programs [1–3]. In mouse, the primitive, or first, wave of hematopoiesis initiates in the yolk sac at about embryonic day 7.5 and produces primarily nucleated embryonic erythrocytes and macrophages [4,5]. The definitive, or second, wave of hematopoiesis is believed to originate from the intra-embryonic aorta–gonads–mesonephros at approximately embryonic day 8.5 and give rise to all the mature blood cell types [6–8].

Similar to that of mammals, zebrafish hematopoiesis also consists of primitive and definitive programs, and produces differentiated cells analogous to most of the mature blood lineages found in mammals [9–11]. Zebrafish primitive erythropoiesis originates from the posterior lateral mesoderm (PLM) as a pair of bilateral stripes at approximately the five-somite stage [9,10,12]. These bilateral stripes extend anteriorly and posteriorly, and converge in the midline at the 20-somite stage to form the main structure of the intermediate cell mass (ICM), where the erythroid progenitors further develop. On the other hand, primitive myelopoiesis is believed to arise from the rostral blood island of the anterior lateral mesoderm (ALM) region at around the ten-somite stage, and produces mainly macrophages [10,13]. Compared to the onset of primitive hematopoiesis, the onset of zebrafish definitive hematopoiesis is less well defined. Preliminary studies indicate that the earliest definitive hematopoietic stem and progenitor cells arise from the ventral wall of dorsal aorta (DA) at around 26 to 30 h postfertilization (hpf) and subsequently migrate to the kidney, the adult hematopoietic organ in zebrafish, by 5 d postfertilization (dpf) [10,14,15].

*Stem-cell leukemia (SCL,* also known as *TAL1)* was originally identified as a proto-oncogene through the study of T cell acute lymphoblastic leukemia patients with a chromosomal translocation at the breakpoint of t(1;14) (p32;q11) [16–18]. The importance of SCL in normal hematopoiesis and angiogenesis was revealed by gene targeting analysis in mouse embryonic stem cells. Mice lacking SCL function failed to form vitelline vessels in the yolk sac and died at embryonic day 8.5 of development because of the complete absence of primitive hematopoiesis [19–21]. SCL-null embryonic stem cells, when injected into blastocysts, failed to contribute to any hematopoietic lineage in mouse chimeras [22,23]. These results demonstrate that SCL is essential for the generation of primitive and definitive hematopoietic cells as well as for the formation of yolk sac vessels. In addition to its pivotal role in early hematopoiesis, SCL also exerts important biological functions in subsequent hematopoietic lineage specification. Enforced *SCL* expression in hematopoietic cell lines favors erythroid differentiation [24,25], and ablation of *SCL* in adult mice impairs erythropoiesis and megakarypoiesis [26,27]. Despite its important functions, the molecular mechanisms of how SCL mediates these multiple functions remain obscure. Previous in vitro studies in human and mouse malignant hematopoietic cell lines have described several SCL isoforms involved in T cell leukemia development and differentiation of erythrocytes and megakaryocytes [28–33]. However, the existence and biological functions of these SCL isoforms in vivo have not been demonstrated.

In this study, we report that the zebrafish produces, through an alternative promoter site within exon 2, a novel *scl (tal1)* isoform, *scl-*β, that encodes a truncated protein lacking the N-terminal 118 amino acids (aa). Whole-mount in situ hybridization (WISH) demonstrated that *scl-*β exhibits temporal and spatial expression that overlaps but is clearly distinctive from that of the full-length *scl-*α: *scl-*β emerges first and expresses in the entire ALM and PLM and the ventral wall of DA, where the first definitive hematopoietic progenitors arise; *scl-*α expresses later in the posterior of the ALM and the PLM, possibly in a manner overlapping with *scl-*β, and is subsequently restricted to the ICM region. Loss-of-function analysis using an antisense morpholino oligonucleotide (MO) knockdown approach revealed that while *scl-*α and -β are redundant in the initiation of primitive hematopoiesis, these two isoforms appear to function at different stages of primitive erythroid cell differentiation. Analysis of definitive hematopoiesis of *scl-*α and -β MO-injected embryos (morphants) showed that the knockdown of Scl-β, but not Scl-α, protein expression resulted in the loss of *c-myb (cmyb)* and *runx1* expression in the ventral wall of DA as well as *rag1* expression in the thymus, demonstrating that *scl-*β but not *scl*-α is essential for definitive hematopoietic stem cell development. Interestingly, we found that *scl-*β and *-*α exhibited a remarkable difference in their protein expression levels both in vitro and in vivo, indicating that differences in the protein expression level of *scl-*α and -β isoforms likely confer their distinct functions in the regulation of hematopoietic cell development.

## Results

### Identification of a Novel Zebrafish *scl* Isoform, *scl-*β

**Figure 1:**
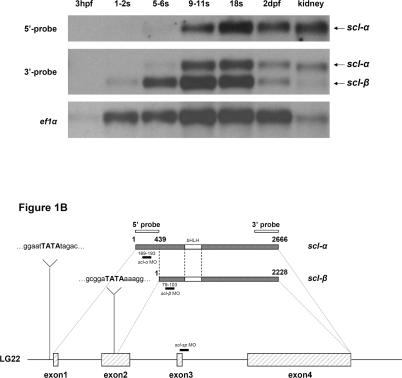
Identification of Zebrafish *scl-*β Isoform (A) Northern blot analysis of *scl* expression in 3-h, 1- to 2-somite (s), 5- to 6-somite, 9- to 11-somite, 18-somite, and 2-dpf embryos and adult kidney. The 3′-probe (3′ UTR) recognized both *scl-*α and -β isoforms (middle), whereas the 5′-probe (5′ UTR) detected only the full-length *scl-*α (top). *ef1*α was used as the loading control (bottom). (B) Gene structures of *scl-*α and -β. *scl-*α consists of 4 exons (boxed regions). *scl-*β transcript is initiated from the middle region of exon 2, and a potential TATA-box sequence (indicated in bold capital letters) is predicted at position −31 of the transcription initiation site. The black bars indicate the positions of *scl-*α MO, *scl-*β MO, and *scl-sp* MO. The white boxes represent sequences that encode the basic helix-loop-helix domain.

To investigate whether different *scl* isoforms exist in zebrafish, RNA samples were prepared from 18-somite-stage embryos and kidney, the adult hematopoietic organ in zebrafish [10], and subjected to Northern blot analysis. The result showed that two transcripts, one 2.6 kilobases (kb) and the other 2.2 kb, were specifically hybridized to the probes corresponding to the coding sequence and the 3′ untranslated region (UTR) of the zebrafish *scl* cDNA (data not shown), suggesting that the 2.6-kb and 2.2-kb transcripts may represent two different *scl* isoforms. To characterize the nature of these two transcripts, we carried out a rapid amplification of cDNA ends (RACE) experiment and obtained one 3′ RACE and two 5′ RACE products (data not shown). DNA sequencing revealed that the larger 5′ RACE product was identical to the published full-length *scl* sequence [34,35], whereas the smaller fragment was also identical except that it lacked the first 438 base pairs at the 5′ end of the full-length *scl,* indicating that the 2.6-kb transcript is the full-length *scl* and the 2.2-kb transcript represents a novel *scl* isoform. This was confirmed by Northern blot analysis, which showed that while both transcripts were hybridized to the *scl* 3′ UTR probe (3′-probe), only the larger 2.6-kb species was recognized by the *scl* 5′ 414-bp probe (5′-probe) (Figure 1A). We hereafter designate the 2.6-kb full-length and the 2.2-kb truncated forms as *scl-*α and *scl-*β, respectively. Time course analysis by Northern blot revealed that the expression of *scl-*β began at the one- to two-somite stage, peaking at around the 11- to 18-somite stage, and maintained a lower level in the adult kidney (Figure 1A). On the other hand, the *scl-*α transcript was not detected until the four-somite stage, after which it rapidly increased its level to that of *scl-*β, subsequently becoming the dominant form in the adult kidney (Figure 1A). Considering the fact that the *scl-*β transcript initiates within the exon 2, where a potential TATA-box sequence can be found at position −31 of the predicted transcription initiation site (Figure 1B), and expresses earlier than *scl-*α (Figure 1A), we conclude that *scl-*β is generated from an alternative promoter site within exon 2 encoding a truncated Scl protein lacking the N-terminal 118 aa (Figure S1).

### Temporal and Spatial Expression Patterns of *scl-*α and -β

**Figure 2:**
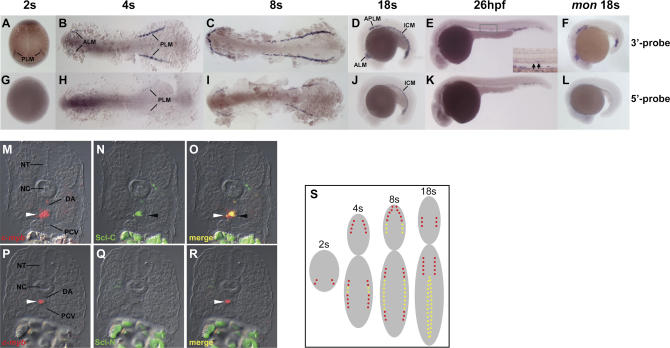
Distinct Expression Pattern of *scl-*α and *-*β during Early Embryonic Development (A–L) Examination of *scl-*α and -β expression by WISH in wild-type and *mon* mutant embryos. WISH was performed in 2-somite (s) (A and G), 4-somite (B and H), 8-somite (C and I), 18-somite (D and J), and 26-hpf (E and K) wild-type embryos, and 18-somite *mon* mutant embryos (F and L). The 3′-probe (A–F) detected the expression of both *scl-*α and -β, whereas the 5′-probe (G–L) detected *scl-*α expression. The arrows indicate the expression of *scl-*β (E), but not *scl-*α (K), in the ventral wall of DA. Embryos are in dorsal (A and G), flat-mounted dorsal (B, C, H, and I), and lateral (D–F and J–L) views with anterior to the top (A and G) or the left (B–F and H–L). (M–R) Transverse sections through the trunk region of 26-hpf wild-type embryos with dorsal up. Fluorescence in situ hybridization showed the expression of *c-myb* in the ventral wall of DA (white arrowhead in [M, O, P, and R]). Immunochemistry staining showed that Ab-Scl-C (black arrowhead in [N and O]) but not Ab-Scl-N (Q) detected Scl expression in the ventral wall of DA, demonstrating the co-localization of *scl-*β and *c-myb* in the ventral wall of DA (O). NC, notochord; NT, neural tube; PCV, posterior cardinal vein. (S) Schematic diagram in flat-mounted dorsal view to illustrate the temporal and spatial expression of *scl-*α and -β. Red represents *scl-*β expression, whereas yellow indicates *scl-*α/β co-expression.

WISH was performed to examine the temporal and spatial expression of *scl-*α and -β. As shown in Figure 2, the 3′-probe, which recognized both *scl-*α and -β, exhibited a pattern identical to that of *scl* expression described previously [34,35]. It first emerged at around the two-somite stage as one pair of stripes in the PLM followed by the appearance of a second pair of stripes in the ALM at the four-somite stage (Figure 2A and 2B). These two pairs of stripes, which represented the combination of both *scl-*α and -β transcripts (referred to as *scl-*α/β stripes hereafter), extended anteriorly and posteriorly from the four-somite stage onwards (Figure 2B and 2C). And by the 18-somite stage, the *scl-*α/β stripes were mainly localized in three regions: the ALM, the anterior of the PLM (APLM), and ICM (Figure 2D). In contrast to the 3′-probe-positive signals, the 5′-probe-positive signals, which represented only the full-length *scl-*α, appeared at the four- and six-somite stage as two pairs of stripes (*scl-*α stripes) in the PLM and ALM, respectively (Figure 2H, and data not shown). By the 18-somite stage, the *scl-*α expression was restricted to the ICM (Figure 2J), where primitive erythropoiesis actively occurs at this stage [10], suggesting that *scl-*α is predominantly expressed in the erythroid lineage from the 18-somite stage onwards. This possibility was further supported by the lack of *scl-*α expression in the erythrocyte-deficient *mon (trim33)* mutant embryos [36] (Figure 2F and 2L). Notably by 26 hpf, only a weak signal of the 3′- but not the 5′-probe was detected in the ventral wall of DA (Figure 2E and 2K), where the first definitive hematopoietic stem cells presumably emerge [10,14,15], suggesting that *scl-*β is the main isoform expressed in the definitive hematopoietic stem cells. To test this possiblity, we carried out double staining analysis in which 26 hpf embryos were stained with anti-Scl-α (Ab-Scl-N) or anti-Scl-α/β (Ab-Scl-C) antibodies together with *c-myb* WISH. The transverse section through the trunk region of these embryos showed that Scl-β but not Scl-α protein was present in the ventral wall of DA in a manner partially overlapping with *c-myb*-positive cells (Figure 2M–2R), indicating that the definitive hematopoietic stem cells predominantly express *scl-*β. Based on these observations, we conclude (as illustrated in Figure 2S) that *scl-*β appears first and expresses in the entire ALM and PLM regions; *scl-*α emerges later in the ALM and ICM, possibly in a manner overlapping with *scl-*β, and is subsequently restricted to the ICM by the 18-somite stage. Notably, only *scl-*β expresses in the ventral wall of DA where the first definitive hematopoietic stem cells arise.

### Redundant Roles for *scl-*α and -β in the Initiation of Primitive Hematopoiesis

**Figure 3:**
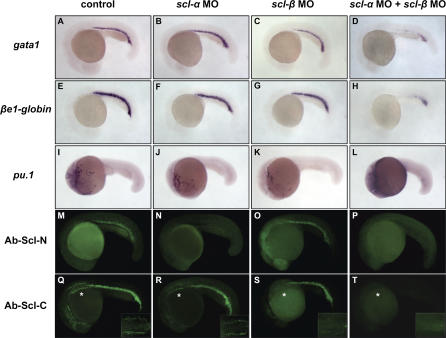
*scl-*α and -β Are Functionally Redundant in the Initiation of Primitive Hematopoiesis (A–L) WISH of *gata1* (A–D), *βe1-globin* (E–H), and *pu.1* (I–L). Similar expression of *gata1, βe1-globin,* and *pu.1* were detected in 20-hpf control embryos (A, E, and I), *scl-*α morphants (B, F, and J), and *scl-*β morphants (C, G, and K). In contrast, expression of *gata1, βe1-globin,* and *pu.1* were significantly reduced or absent in *scl-*α*/*β co-injected morphants (D, H, and L). All embryos are in lateral view with anterior to the left. (M–T) Immunohistochemistry staining of endogenous Scl proteins in 20-hpf control embryos (M and Q) and *scl-*α (N and R), *scl-*β (O and S), and *scl-*α*/*β co-injected (P and T) morphants using Ab-Scl-N (M–P) and Ab-Scl-C (Q–T). In *scl-*α morphants, the Ab-Scl-N (N), but not Ab-Scl-C (R), staining in the ICM was absent, showing that *scl-*α MO specifically blocks the translation of *scl-*α. In the *scl-*β morphants, the Ab-Scl-C staining was selectively abolished in the APLM, where only *scl-*β is transcribed ([S] asterisk, inset), demonstrating that *scl-*β MO specifically blocks the protein expression of *scl-*β. In the *scl-*α*/*β co-injected morphants, neither Ab-Scl-N nor Ab-Scl-C staining was detected (P and T). Insets in (Q–T) are dorsal views of the magnified APLM marked by asterisks. Embryos are in lateral view with anterior to the left.

To determine the biological functions of *scl-*α and -β in hematopoiesis, two MOs, *scl-*α MO and *scl-*β MO (Figure 1B), that specifically inhibited the protein syntheses of *scl-*α and -β, respectively, were injected into wild-type zebrafish embryos. Immunohistochemistry staining showed that *scl-*α MO and -β MO specifically abolished the Scl-α and -β protein expression as indicated by lack of anti-Scl-N staining in the ICM of *scl-*α morphants (Figure 3N, *n =* 35/35), and the selective loss of anti-Scl-C staining in the APLM (where only *scl-*β was transcribed [Figure 2D]) of *scl-*β morphants (Figure 3S, *n =* 32/32). Microscopic analysis of the 24-hpf embryos injected with either MO revealed no obvious abnormality in general morphology (data not shown). Examination of the expression of *gata1* (Figure 3A, *n =* 46/46; 3B, *n =* 43/43; and 3C, *n =* 41/41), β*e1-globin (hbbe1)* (Figure 3E, *n =* 49/49; 3F, *n =* 51/51; and 3G, *n =* 49/49), and *pu.1 (spi1)* (Figure 3I, *n =* 41/41; 3J, *n =* 41/43; and 3K, *n =* 45/45) by WISH confirmed that the initiation of primitive erythropoiesis and myelopoiesis were intact in the *scl-*α and -β morphants. We reasoned that the lack of phenotypes in both morphants was likely due to the functional redundancy of *scl-*α and -β. To test this possibility, we co-injected *scl-*α MO and *scl-*β MO to block both protein syntheses (Figure 3P, *n =* 45/45; and 3T, *n =* 50/50), and found that the expression of *gata1, βe1-globin,* and *pu.1* were either absent or drastically reduced in the co-injected morphants (Figure 3D, *n =* 42/42; 3H, 46/46; and 3L, *n =* 47/47). This phenotype is very similar to that found in the *scl-sp* morphants, in which both Scl-α and -β protein expression were eliminated by *scl-sp* MO (Figure 1B) that interfered with the splicing between exon 2 and 3 of the *scl* gene [37,38]. Furthermore, injection of in vitro synthesized *scl-*α or -β mRNA was sufficient to rescue the expression of *gata1, βe1-globin,* and *pu.1* in the *scl-sp* morphants (data not shown). Taken together, we conclude from these results that *scl-*α and -β are functionally redundant in the initiation of primitive hematopoiesis.

### *scl-*α and -β Play Distinct Roles in Primitive Red Blood Cell Differentiation

**Figure 4:**
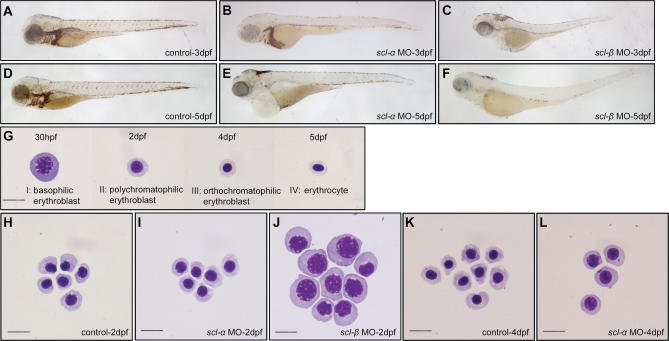
Requirement of *scl-*α and -β at Different Development Stages of Primitive RBC Differentiation (A–F) O-dianisidine staining of hemoglobin. At 3 dpf, expression of hemoglobin was severely reduced in *scl-*β morphants (C) compared to control embryos (A) and *scl-*α morphants (B). At 5 dpf, the o-dianisidine staining in both *scl-*α (E) and *scl-*β (F) morphants was dramatically decreased compared to that in control embryos (D). All embryos are in lateral view with anterior to the left. (G–L) May-Grunwald Giemsa staining of RBCs of different developmental stages (30 hpf to 5 dpf). Normal primitive RBCs at 30 hpf to 5 dpf can be classified into four main developmental stages (G): stage I, basophilic erythroblast; stage II, polychromatophilic erythroblast; stage III, orthochromatophilic erythroblast; and stage IV, mature erythrocyte. While RBCs from 2-dpf control embryos (H) and *scl-*α morphants (I) have differentiated into the polychromatophilic erythroblast stage (stage II), RBCs from *scl-*β morphants (J) are halted at the earlier basophilic erythroblast stage (stage I). At 4 dpf, RBCs from *scl-*α morphants (L) are arrested at stage II, while there is normal development of stage IV RBCs in control embryos (K). Scale bar (G–L), 10 μm.

**Figure 5:**
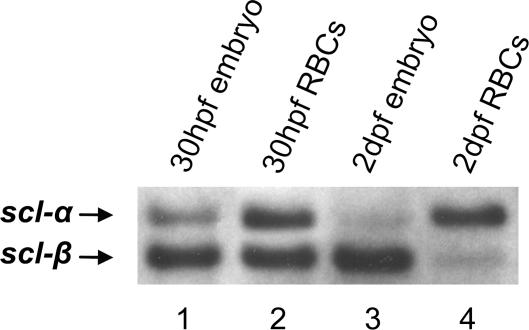
The *scl-*α and -β Transcripts in 30-hpf and 2-dpf Embryos and RBCs Virtual Northern blotting shows that 30-hpf RBCs express both *scl-*α and -β (lane 2) whereas 2-dpf RBCs express predominantly *scl-*α (lane 4).

To address their functions in the late developmental stages of primitive erythropoiesis, we examined the *scl-*α and -β morphants beyond 30 hpf. Although primitive erythropoiesis initiated normally, o-dianisidine staining revealed that the red blood cells (RBCs) in the *scl-*β morphants were significantly reduced by 2 dpf (data not shown) and finally not detectable by 3 dpf (Figure 4C). On the other hand, RBCs in the *scl-*α morphants were normal before 3 dpf (Figure 4B) but began to decrease by 4 dpf (data not shown) and were severely reduced by 5 dpf (Figure 4E). These data indicate that the loss of either Scl-α or -β protein renders abnormal RBC differentiation at different developmental stages, eventually resulting in anemia. To provide an additional test of this possibility, circulating RBCs were collected from the *scl-*α and -β morphants, stained with May-Grunwald Giemsa, and compared to those from wild-type embryos. Based on the size of cell, shape of nucleus, and staining of cytoplasm, normal primitive RBCs from 30 hpf to 5 dpf can be classified into four main stages: stage I, basophilic erythroblast; stage II, polychromatophilic erythroblast; stage III, orthochromatophilic erythroblast; and stage IV, mature erythrocyte (Figure 4G). In the 2-dpf wild-type embryos and 2-dpf *scl-*α morphants, more than 98% of the circulating RBCs were at stage II (Figure 4H and 4I). However, 90% of the circulating RBCs arrested at stage I in the 2-dpf *scl-*β morphants (Figure 4J). As expected, although the circulating RBCs developed normally before 3 dpf, 80% of them were blocked at stage II in the 4-dpf *scl-*α morphants (Figure 4L), compared to the number of RBCs at stage III in the 4-dpf wild-type embryos (Figure 4K). These phenotypes concurred with the virtual Northern blot result showing that circulating RBCs from 30-hpf wild-type embryos contain both *scl-*α and -β, whereas those from 2-dpf wild-type embryos express predominantly *scl-*α (Figure 5). Collectively, we conclude that *scl-*β plays a critical role in the differentiation of basophilic erythroblasts to polychromatophilic erythroblasts, whereas *scl-*α is pivotal for the transition from polychromatophilic erythroblasts to orthochromatophilic erythroblasts.

### Specification of Definitive Hematopoietic Stem Cells Requires *scl-*β but Not -α

**Figure 6:**
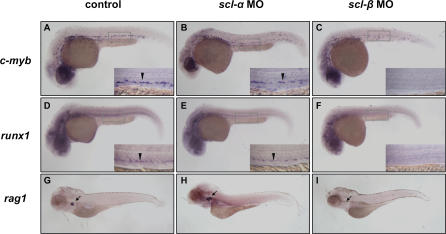
Specification of Definitive Hematopoietic Stem Cells Requires *scl-*β but Not *scl-*α (A–F) WISH of definitive hematopoietic stem cell markers *c-myb* (A–C) and *runx1* (D–F). Expression of *c-myb* and *runx1* in the ventral wall of DA was markedly reduced in 30-hpf *scl-*β morphants (C and F) but not in 30-hpf control embryos (A and D) or *scl-*α morphants (B and E). Insets are high-magnification (20×) views of the ventral wall of DA. The arrowheads indicate the definitive hematopoietic stem cells along the ventral wall of DA. (G–I) WISH of T cell marker *rag1*. *rag1* expression in thymic T cells was abolished in 5-dpf *scl-*β morphants (I) but not in control embryos (G) and *scl-*α morphants (H). Arrows indicate the thymus region. In all panels, embryos are in lateral view with anterior to the left.

We next explored the roles of the *scl*-β and -α isoforms in definitive hematopoiesis. In zebrafish, definitive hematopoietic stem cells originate from the ventral wall of DA at around 26 hpf to 30 hpf, and these cells are enriched in *c-myb* and *runx1* expression [10,14,15]. We therefore first examined *c-myb* and *runx1* expression in the 30-hpf *scl-*α and -β morphants. WISH revealed that expression of *c-myb* and *runx1* in the ventral wall of DA was abolished in the *scl-*β morphants (Figure 6C, *n =* 43/45; and 6F, *n =* 40/43) but not in the control embryos (Figure 6A, *n =* 40/40; and 6D, *n =* 42/42) or *scl-*α morphants (Figure 6B, *n =* 45/46; and 6E, *n =* 43/45). As artery endothelial cells appeared to be retained in both *scl-*α and -β morphants, as indicated by two artery-specific markers *deltaC (dlc)* and *grl (hey2)* (Figure S2), these data indicate that *scl-*β*,* but not -α, is essential for definitive hematopoietic stem cell development. This result is consistent with the finding that only *scl-*β is expressed in the ventral wall of DA (Figure 2). To further test whether *scl-*β is indeed required for the development of definitive hematopoietic stem cells, we investigated T cell development in both morphants by examining *rag1* expression at 5 dpf. As expected, *rag1* was detected in the thymus of control embryos and *scl-*α morphants (Figure 6G, *n =* 30/30; and 6H, *n =* 42/45) but not *scl-*β morphants (Figure 6I, *n =* 46/50). Taken together, these data demonstrate that *scl-*β is essential for the development of definitive hematopoietic stem cells while *scl-*α is dispensable.

### Differences in the Protein Expression Level of Scl-α and -β Give Rise to Their Distinct Functions

**Figure 7:**
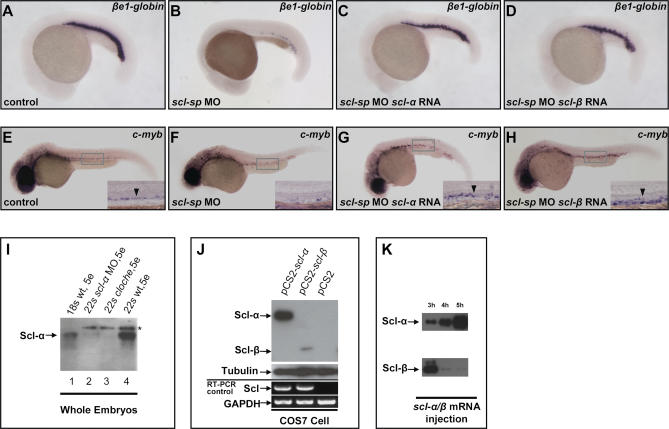
Differences in Protein Expression Levels of Scl-α and -β Confer Their Distinct Functions (A–H) β*e1-globin* and *c-myb* expression in *scl-sp* morphants is rescued by *scl-*α and *-*β mRNA. (A–D) show WISH of β*e1-globin* in 20-hpf control embryos (A), *scl-sp* morphants (B), *scl-sp* morphants injected with *scl-*α mRNA (C), and *scl-sp* morphants injected with *scl-*β mRNA (D). (E–H) show WISH of *c-myb* in 30-hpf control embryos (E), *scl-sp* morphants (F), *scl-sp* morphants injected with *scl-*α mRNA (G), and *scl-sp* morphants injected with *scl-*β mRNA (H). Insets in (E–H) are high-magnification (20×) views of the ventral wall of DA, indicated by black arrowhead. In all panels, embryos are in lateral view with anterior to the left. (I) Immunoblotting with Ab-Scl-C antiserum shows the expression levels of Scl-α and Scl-β proteins in wild-type (wt) embryos, *clo* mutant embryos, and *scl-*α morphants. Although Scl-β protein was not detectable, immunoblotting of whole embryo protein extracts (50 μg for each sample) showed that Scl-α protein expression increased as embryos developed from the 18-somite (lane 1) to 22-somite (lane 4) stage. The *clo* mutant embryos (lane 3) and *scl-*α morphants (lane 2), in which Scl-α protein was not detected, were used as the controls to distinguish the Scl-α protein (arrow) and nonspecific band (asterisk). (J) Immunoblotting with Ab-Scl-C antiserum shows the expression levels of Scl-α and Scl-β proteins in transfected COS7 cells. Western blotting of whole cell protein extracts (10 μg for each sample) prepared from COS7 cells transfected with full-length *scl-*α, *scl*-β, or blank vector constructs revealed that the protein level of Scl-α was much higher than that of Scl-β. RT-PCR analysis (bottom) showed similar RNA levels of *scl-*α and -β in these transfected cells. Tubulin and GAPDH were used as controls for Western blot and PCR, respectively. (K) Immunoblotting with Ab-Scl-C antiserum shows the expression levels of Scl-α and Scl-β proteins in the embryos injected with in vitro synthesized *scl-*α or -β mRNA. Western blotting of whole embryo protein extracts (50 μg for each sample) from embryos injected with 500 pg of *scl-*α or *-*β mRNA showed that, at 3 h post-injection, the Scl-β protein was detected as being at a comparable level to that of Scl-α but dramatically reduced by 4 h post-injection.

To gain insight into the molecular basis underlying the distinct functions of *scl-*α and -β in hematopoietic cell development, we performed rescue experiments—by co-injecting in vitro synthesized *scl-*α or *scl-*β mRNA with *scl-sp* MO into wild-type embryos—to test whether the lack of the N-terminal 118 aa could lead to the differences in their biological functions. Examination of the expression of *βe1-globin* and *c-myb* by WISH showed that either isoform was sufficient to rescue both primitive and definitive hematopoietic defects in the *scl-sp* morphants (Figure 7A, *n =* 35/35; 7B, *n =* 41/41; 7C, *n =* 44/44; 7D, *n =* 47/47; 7E, *n =* 36/36; 7F, *n =* 40/45; 7G, *n =* 41/45; and 7H, *n =* 42/46). The data indicate that the N-terminal 118-aa segment is not essential for determination of the functions of these two Scl protein isoforms, consistent with previous findings showing that the basic helix-loop-helix domain of the murine SCL protein is sufficient for hematopoiesis [39]. As recent studies have suggested that different SCL protein expression levels are required at different levels of hematopoietic hierarchy [40,41], these findings raise the possibility that the distinct functions of *scl-*α and -β are due to differences in their protein expression levels, resulting in establishment of a gradient of Scl protein at different stages of hematopoietic hierarchy. To test this hypothesis, we performed immunoblotting of protein extracts from 18-somite-stage wild-type embryos, in which the transcription levels of the *scl-*α and -β isoforms are similar (the *scl-*β RNA is slightly higher) (Figure 1A). We found that the protein expression level of Scl-β was much lower than that of Scl-α in these embryos (the Scl-β protein was hardly detectable at all by Western blot; Figure 7I). When COS7 cells were transiently transfected with construct expressing either the full-length *scl-*α or *scl-*β, the protein expression level of Scl-β was also much lower than that of Scl-α, while their RNA levels were comparable (Figure 7J). These data strongly indicate that a post-transcriptional mechanism is involved in the regulation of Scl-β protein expression level. To test this, equal amounts of in vitro synthesized *scl-*α and -β mRNA were injected into one-cell-stage wild-type embryos, and protein levels were examined at different time points post-injection (Figure 7K). As anticipated, real-time reverse transcriptase PCR analysis revealed that mRNAs of both isoforms behaved similarly in these injected embryos (data not shown). Immunoblotting of whole embryo protein extracts showed that protein expression levels were comparable at 3 h post-injection, indicating that both isoforms are effectively translated. However, by 4 h post-injection, Scl-β protein level was greatly reduced, while Scl-α level increased (Figure 7K), showing that the low protein expression level of Scl-β was likely due to the rapid degradation of its protein. Taken together, these data strongly indicate that differences in the protein expression levels of Scl-α and -β isoforms, via regulation of their protein stabilities, likely confer their distinct functions in the regulation of hematopoietic cell development.

## Discussion

It is believed that the complexity in morphology and behavior of higher organism is achieved not only by higher gene numbers, but also by multiple protein isoforms being encoded by a single gene locus and by the complexity of protein–protein interactions. The most well studied phenomenon that results in the generation of multiple protein isoforms from a single gene is alternative splicing of pre-mRNA [42]. However, other mechanisms such as use of an alternative promoter—a phenomenon that is as equally widespread in higher organisms as alternative pre-mRNA splicing [43,44]—are less appreciated. In this article, we described how zebrafish produce, through alternative promoter sites, two *scl* isoforms, the full-length *scl-*α and a novel truncated *scl-*β (Figure 1). We further showed that these two *scl* isoforms manifest distinct temporal and spatial expression (Figure 2) and exert distinct functions in the regulation of primitive and definitive hematopoiesis (Figures 3–6). The identification of the alternative-promoter-generated *scl-*β isoform in zebrafish has not only revealed new insight into the roles of *scl* in the regulation of hematopoietic cell development, but also provided another example to highlight the importance of alternative promoter usage in generating protein and regulatory diversity.

Previous studies have revealed that mammals contain several *SCL* isoforms generated by either alternative splicing, alternative promoters, or alternative translation initiation sites [28–33]. These mammalian *SCL* isoforms arise from alternative promoters in exon 1a and exon 1b, and encode identical proteins, the full-length SCL. In contrast, *scl-*β in zebrafish is generated through an alternative promoter site within the *scl* exon 2, the equivalent of the mammalian *SCL* exon 4, and encodes an N-terminal truncated protein (Figures 1 and S1). Thus, *scl-*β is clearly distinct from the previously described mammalian *SCL* isoforms. One intriguing question raised is whether the mechanism of generating different *scl* isoforms found in zebrafish is evolutionarily conserved in higher vertebrates, especially in mammals. Considering the facts that (1) the *SCL* locus is highly conserved in vertebrates [45], (2) there are observations showing that the murine *SCL* exon 4 has promoter activity in the context of *SCL* 3′ stem cell enhancer [46], and (3) a truncated *SCL* transcript initiated from exon 4 can be detected in some of the human T cell leukemia cell lines [28,29], we speculate that a *scl-*β equivalent may exist in higher vertebrate species.

Another interesting issue raised by this study is the regulation of *scl-*α and -β transcripts during hematopoiesis. Notably, *scl-*β first appears in hematopoietic stem and progenitor cells and soon diminishes in the differentiated primitive RBCs (Figures 2 and 5). In contrast, *scl-*α emerges later and is predominantly restricted to RBCs (Figures 2 and 5). Thus, it appears that an on–off switch, from *scl-*β to -α expression, must occur during primitive RBC development. Considering the facts that (1) the earliest definitive hematopoietic stem/progenitor cells located in the ventral wall of DA express only *scl-*β and (2) *scl-*α becomes the predominant isoform expressed in the adult kidney marrow, where definitive hematopoiesis takes place presumably from 5 dpf onwards in zebrafish development, it is conceivable to speculate that this on–off switch may also exist during definitive erythroid cell development. However, it is unclear at this moment whether this on–off switch takes place at the transcriptional level or the post-transcriptional level, or perhaps a combination of both. Nevertheless, we believe that the on–off switch of these *scl* isoforms must play a crucial role in normal hematopoietic cell development, at least for RBCs, and that the underlying molecular basis of this regulation warrants further studies.

Our study has provided evidence indicating that differences in the protein expression levels of the *scl-*α and -β isoforms are likely to confer their distinct functions in regulating hematopoietic cell development (Figure 7). Although we cannot rule out the possibility that translational control may contribute to the regulation of their protein expression levels, the fact that the Scl-β protein was initially expressed at a level comparable to that of Scl-α but soon reduced dramatically upon injection of equal amounts of in vitro synthesized *scl-*α and -β mRNA (Figure 7K) strongly indicates that differences in the protein expression levels of the *scl-*α and -β isoforms are largely due to the rapid turnover of the Scl-β protein. However, the triggers causing the onset of rapid degradation of Scl-β protein in vivo are unclear. Given the differences in their N-terminal residues, one could speculate that, perhaps, the short half-life of the Scl-β protein is mediated through the N-end rule degradation, a common proteolytic pathway that is present in prokaryotes, fungi, plants, and animals [47]. Further biochemical analyses are required to clarify this issue.

Finally, our data strongly suggest that the establishment of an appropriate Scl protein gradient at different levels of hematopoietic hierarchy—a low level in hematopoietic stem and progenitor cells and a high level in differentiated RBCs—is essential for hematopoietic cell development. The phenomenon of a lower Scl protein level in hematopoietic stem and progenitor cells correlating to the importance of hematopoietic cell development is intriguing. One possibility is that the specification of definitive stem and progenitor cells requires the low concentration of Scl protein, which occurs by preferential expression of *scl-*β during early hematopoiesis. However, the fact that injection of either *scl-*α or -β mRNA is sufficient to rescue the *c-myb* expression at 30 hpf in the ventral wall of DA in *scl-sp* morphants (Figure 7) suggests that this may not be the case. A high Scl protein level, which is known to be required for the maturation of RBCs [40,41], has the tendency to promote hematopoietic stem and progenitor cell differentiation into erythroid lineage, so a more likely explanation is that the low concentration of the Scl-β protein ensures the proper expansion of these cell pools by promoting their proliferation rather than their differentiation. In addition, the low concentration of Scl-β may also be crucial for maintaining an unbiased differentiation potential of hematopoietic stem and progenitor cells during ontogeny. Uncovering the molecular basis of *scl-*α- and -β-mediated actions will provide further insight into our understanding of the specification, proliferation, and differentiation of hematopoietic lineages.

## Materials and Methods

### Zebrafish.

Zebrafish were maintained at 27 to 28 °C as described in [48]. The *clo* ^s5^ and *mon* ^tg234^ mutants were kindly provided by Didier Y. R. Stainier (University of California San Francisco, United States) and Artemis Pharmaceuticals (Germany), respectively.

### *scl* plasmid constructs.

The full-length *scl-*α and -β DNA were amplified by RT-PCR and cloned into pCS2+ vector. The *scl*-5′ and *scl*-3′ constructs contained the first 414 bp of the 5′ UTR of *scl-*α and the last 539 bp of the 3′ UTR of *scl-*α/β, respectively. They were amplified by PCR using two sets of specific primers (*scl*-5′: 5′-acttcagtgcatctaaaacctcag-3′/5′-ttttatatccgcgctccctcctc-3′; *scl*-3′: 5′-tggaaattcaagcgggtaatgac-3′/5′-gggcttttcatataaaatttgtgag-3′) and subcloned into pGEM-T Easy and pGEM-T vector (Promega, http://www.promega.com).

### 5′ and 3′ RACE.

Total RNA was extracted from wild-type embryos and subjected to 5′ and 3′ RACE using the SMART RACE cDNA Amplification Kit (Clontech, http://www.clontech.com) according to the manufacturer's instructions. For 5′ and 3′ RACE, two sets of primers (*scl*-5′-P1: 5′-aagttgatgtacttcatggccag-3′; *scl*-5′-P2: 5′-atacatcccatactgttccgcatctccagc-3′; *scl*-3′-P1: 5′-gcggaacagtatgggatgtatcct-3′; *scl*-3′-P2: 5′-ctagtgcgggacgacctc-3′) were used. The RACE products were cloned into pGEM-T Easy vector (Promega) and subsequently sequenced.

### Sequence alignment analysis.

The T-Coffee method [49] was used for protein sequence alignment.

### Northern and virtual Northern blot.

Total RNA from different stages of embryos, adult kidney, and RBCs were prepared using the RNeasy Kit (Qiagen, http://www.qiagen.com) according to the manufacturer's instructions. mRNA was purified using the NucleoTrap mRNA mini kit (Macherey-Nagel, http://www.macherey-nagel.com) according to the manufacturer's instructions. Three micrograms of each embryonic mRNA and 30 μg of total kidney RNA were used for Northern blot analysis (Figure 1A). For virtual Northern blot, total RNA from 30-hpf and 2-dpf embryos and RBCs were reverse transcribed into cDNA and then amplified by the SMART PCR cDNA synthesis kit (Clontech). The amplified cDNAs were used as targets for hybridization with the DIG-labeled *scl*-5′ and *scl*-3′ probes. DIG labeling was carried out by PCR amplification with the DIG Probe Synthesis Kit (Roche Applied Science, http://www.roche-applied-science.com) according to the manufacturer's instructions. Northern blot and virtual Northern blot analyses were performed as previously described [50,51].

### O-dianisidine staining.

Fish embryos were stained for 15 min in the dark in o-dianisidine staining solution as previously described [12].

### Whole-mount in situ hybridization.

Generation of the DIG-labeled anti-sense RNA probes and whole-mount in situ hybridization were performed as described in [48]. For transverse cryosection, embryos were embedded in OCT solution (Sakura, http://www.sakura.com) after WISH and immunochemistry staining. Embedded embryos were sectioned at a thickness of 10 μm using a Leica (http://www.leica.com) microtome. The sections were mounted on glass slides and imaged using a Zeiss (http://www.zeiss.com) AxioPhot 2 imaging system.

### Immunohistochemistry analysis.

Anti-zebrafish Scl-α N-terminal (aa 17 to 62, referred to as Ab-Scl-N) and Scl-α/β C-terminal (aa 255 to 325, referred to as Ab-Scl-C) antisera were generated by immunizing rabbits with the GST-Scl-N and GST-Scl-C fusion proteins using standard protocol. Antibody purification and immunohistochemistry staining were carried out as previously described [52].

### MO injection.

Anti-sense MOs (Gene Tools) were designed as follows: *scl-*α MO, 5′-gctcggatttcagtttttccatcat-3′; *scl-*β MO, 5′-gcggactcaactgcaccattcgagt-3′; *scl-sp* MO, 5′-agatttaaaatgctcttaccatcgt-3′. Three nanograms of *scl-*α MO, 8 ng of *scl-*β MO, and 8 ng of *scl-sp* MO mixed with phenol red were separately injected into wild-type embryos at the one-cell stage. Similarly, 3 ng of *scl-*α MO and 8 ng of *scl-*β MO were used for the co-injection experiment. Wild-type embryos injected with a mixture of sterile water and phenol red were used as control.

### May-Grunwald Giemsa staining of embryonic RBCs.

Fish embryos were anesthetized in calcium- and magnesium-free PBS (pH 7.4) containing 0.02% tricaine (Sigma-Aldrich, http://www.sigmaaldrich.com) and 1% BSA (Sigma-Aldrich). After tail clipping using surgical scissors, blood cells were collected by pipetting and cytospun onto slides by centrifugation at 450 rpm for 3 min using a Cytospin 4 (Thermo Scientific, http://www.thermo.com). The slides were then air-dried and subjected to May-Grunwald Giemsa staining according to the standard protocol.

### In vitro RNA synthesis and micro-injection.

In vitro transcription was carried out using the mMESSAGE mMACHINE sp6 kit (Ambion, http://www.ambion.com) according to the manufacturer's instructions. For rescue experiments, 200 pg of *scl-*α or *-*β RNA was co-injected with 8 ng of *scl-sp* MO into wild-type embryos at the one-cell stage.

### Protein stability analysis.

For protein stability analysis in fish embryos, 500 pg of in vitro synthesized *scl-*α or *-*β mRNA was injected into embryos at the one-cell stage, and protein extracts were prepared as described in [48]. Total RNA from both *scl-*α- and *-*β-injected embryos at 6 h post-injection were extracted and reverse transcribed using random hexamer as primer. The cDNAs were examined with real-time PCR using *scl-*specific primers (*scl*-961F, 5′-ctagtgcgggacgacctc-3′; *scl*-1528R, 5′-ggaactaaactgtgccga-3′), which can amplify both injected *scl-*α and *-*β mRNA. COS7 cells were maintained in DMEM (Gibco, http://www.invitrogen.com/content.cfm?pageid=11040) supplemented with 10% bovine calf serum (Hyclone, http://www.hyclone.com). Transient transfection was carried out by SuperFect Reagent (Qiagen) according to the manufacturer's protocol. Real-time RT-PCR was performed to ensure that the transfection efficiency was similar using the same RT-PCR protocol for protein stability analysis in fish embryos. Cell extract preparation and Western blot were carried out as described previously [50].

## Supporting Information

Figure S1 Sequence Alignment of Zebrafish Scl-α/β and Human and Mouse Full-Length SCL Proteins The full-length *scl-*α encodes a protein of 324 aa, whereas *scl-*β encodes a truncated protein lacking the N-terminal 118 aa. The predicted translation start site (indicated by arrow) of *scl-*β is conserved between zebrafish, human, and mouse. The basic helix-loop-helix domain is highlighted in red.
(33 KB DOC)

Figure S2 The Artery Endothelial Cells Are Retained in Both *scl-*α and -β Morphants WISH of artery-specific markers *deltaC* ([A], *n =* 42/42; [B], *n =* 41/44; and [C], *n =* 45/46) and *grl* ([D], *n =* 40/40; [E], *n =* 45/46; and [F], *n =* 44/47). At 24 hpf, expression of *deltaC* and *grl* was only slightly reduced in both *scl*-α ([B and E], arrowheads) and -β morphants ([C and F], arrowheads) when compared to the control embryos ([A and D], arrowheads).
(5.0 MB TIF)

## Abbreviations

aa: amino acid(s)
ALM: anterior lateral mesoderm
APLM: anterior of the posterior lateral mesoderm
DA: dorsal aorta
dpf: days postfertilization
hpf: hours postfertilization
ICM: intermediate cell mass
kb: kilobase(s)
MO: morpholino oligonucleotide
morphant: morpholino oligonucleotide–injected embryo
PLM: posterior lateral mesoderm
RACE: rapid amplification of cDNA ends
RBC: red blood cell
RT-PCR: reverse transcriptase PCR
SCL: stem-cell leukemia
UTR: untranslated region
WISH: whole-mount in situ hybridization
