# Supplementary material for: Distinct Functions for Different scl Isoforms in Zebrafish Primitive and Definitive Hematopoiesis
Source: PLoS Biol. 2007 May 1;5(5):e132. doi: 10.1371/journal.pbio.0050132 (PMC1858710; doi:10.1371/journal.pbio.0050132)
Supplement: Supplementary file 1 [file pbio.0050132.sg001.doc]

```
D. rerio α      MEKLKSEQFPLSPSAEGCASPPRGDGDARGKQEGTTAETGEHRLPEE--LNGVAKETAHH
D. rerio β      ------------------------------------------------------------
H. sapien       -----MTER-----------PPSEAARSDPQLEGRDAAEASMAPPHLVLLNGVAKETSRA
M. musculus     -----MTER-----------PPSEAARSDPQLEGQDAAEARMAPPHLVLLNGVAKETSRA


D. rerio α      ATELKKEVAVIELSRRGGSA-----------DIKGREL-KAELSHKVQTTELCRPPIPLP
D. rerio β      ------------------------------------------------------------
H. sapien       AAA---EPPVIELGARGGPGGGPAGGGGAARDLKGRDAATAEARHRVPTTELCRPPGPAP
M. musculus     APA---EPPVIELGARSGAGGGPASGGGAARDLKGRDAVAAEARLRVPTTELCRPPGPAP

                              Scl-β
```

```
D. rerio α      LPPR-----DPLSDTRMVQLSPPAFPLPA---RAMLYSNMTTPLATINSGFAGDAEQYGM
D. rerio β      ----------------MVQLSPPAFPLPA---RAMLYSNMTTPLATINSGFAGDAEQYGM
H. sapien       APAPASVTAELPGDGRMVQLSPPALAAPAAPGRALLYS-LSQPLASLGSGFFGEPDAFPM
M. musculus     APAPASAPAELPGDGRMVQLSPPALAAPAGPGRALLYS-LSQPLASLGSGFFGEPDAFPM
                                ********:. **   **:*** :: ***::.*** *:.: : *

D. rerio α      YPSN-RVKRRPAPYEVEINDGSQPKIVRRIFTNSRERWRQQNVNGAFAELRKLIPTHPPD
D. rerio β      YPSN-RVKRRPAPYEVEINDGSQPKIVRRIFTNSRERWRQQNVNGAFAELRKLIPTHPPD
H. sapien       FTTNNRVKRRPSPYEMEITDGPHTKVVRRIFTNSRERWRQQNVNGAFAELRKLIPTHPPD
M. musculus     FTNNNRVKRRPSPYEMEISDGPHTKVVRRIFTNSRERWRQQNVNGAFAELRKLIPTHPPD
                :..* ******:***:**.**.:.*:**********************************

D. rerio α      KKLSKNEILRLAMKYINFLAKLLNDQDDMVGGE------APARANRD--SRDATLVRDDL
D. rerio β      KKLSKNEILRLAMKYINFLAKLLNDQDDMVGGE------APARANRD--SRDATLVRDDL
H. sapien       KKLSKNEILRLAMKYINFLAKLLNDQEEEGTQRAKTGKDPVVGAGGGGGGGGGGAPPDDL
M. musculus     KKLSKNEILRLAMKYINFLAKLLNDQEEEGTQRAKPGKDPVVGAGGG--GAGGGIPPEDL
                **************************::    .      . . *. .  . ..    :**

D. rerio α      LQEMLSPNSSCGSLLDGDASPESFTEDQDSSVESRPSARGLHHSSLPL-DGNAQR
D. rerio β      LQEMLSPNSSCGSLLDGDASPESFTEDQDSSVESRPSARGLHHSSLPL-DGNAQR
H. sapien       LQDVLSPNSSCGSSLDGAASPDSYTEEPA----PKHTARSLHPAMLPAADGAGPR
M. musculus     LQDVLSPNSSCGSSLDGAASPDSYTEEPT----PKHTSRSLHPALLPAADGAGPR
                **::********* *** ***:*:**:      .: ::*.** : **  ** . *
```
